# The impact of MRI sequence on tumour staging and gross tumour volume delineation in squamous cell carcinoma of the anal canal

**DOI:** 10.1007/s00330-017-5133-0

**Published:** 2017-11-13

**Authors:** Davide Prezzi, Ramin Mandegaran, Sofia Gourtsoyianni, Katarzyna Owczarczyk, Andrew Gaya, Robert Glynne-Jones, Vicky Goh

**Affiliations:** 1 Division of Imaging Sciences & Biomedical Engineering, King’s College London, 4th Floor, Lambeth Wing, St. Thomas’ Hospital, Westminster Bridge Road, London, SE1 7EH UK; 2 Clinical Imaging, Guy’s and St Thomas’ NHS Foundation Trust, 1st Floor, Lambeth Wing, St. Thomas’ Hospital, Westminster Bridge Road, London, SE1 7EH UK; 3 Imaging 2, Level 1, Lambeth Wing, St Thomas’ Hospital, Westminster Bridge Road, London, SE1 7EH UK; 4 Clinical Oncology, Guy’s and St Thomas’ NHS Foundation Trust, Lambeth Wing, St. Thomas’ Hospital, Westminster Bridge Road, London, SE1 7EH UK; 5 Mount Vernon Cancer Centre, East and North Herts NHS Trust, Rickmansworth Rd, Northwood, HA6 2RN UK

**Keywords:** Anus neoplasms, Magnetic resonance imaging, Neoplasm staging, Radiotherapy, image-guided, Diffusion magnetic resonance imaging

## Abstract

**Objectives:**

To compare maximum tumour diameter (MTD) and gross tumour volume (GTV) measurements between T_2_-weighted (T_2_-w) and diffusion-weighted (DWI) MRI in squamous cell carcinoma of the anal canal (SCCA) and assess sequence impact on tumour (T) staging. Second, to evaluate interobserver agreement and reader delineation confidence.

**Methods:**

The staging MRI scans of 45 SCCA patients (25 females) were assessed retrospectively by two independent radiologists (0 and 5 years’ experience of anal cancer MRI). MTD and GTV were delineated on both T_2_-w and high-b-value DWI images and compared between sequences; T staging was derived from MTD. Interobserver agreement was assessed and delineation confidence scored (1 to 5) by each observer.

**Results:**

GTV and MTD were significantly and systematically lower on DWI versus T_2_-w sequences by 14.80%/9.98% (MTD) and 29.70%/12.25% (GTV) for each reader, respectively, causing T staging discordances in approximately a quarter of cases. Bland-Altman limits of agreement were narrower and intraclass correlation coefficients higher for DWI. Delineation confidence was greater on DWI: 40/42 cases were scored confidently (4 or 5) by each reader, respectively, versus 31/36 cases based on T_2_-w images.

**Conclusions:**

Sequence selection affects SCCA measurements and T stage. DWI yields higher interobserver agreement and greater tumour delineation confidence.

***Key Points*:**

• *MTD and GTV measurements are significantly lower on DWI than on T*_*2*_ *-w MRI*.

• *Such differences cause T staging discordances in up to a quarter of cases*.

• *DWI results in higher agreement between inexperienced and experienced observers*.

• *DWI offers greater tumour delineation confidence to inexperienced readers*.

## Introduction

The incidence of squamous-cell carcinoma of the anus (SCCA), commonly referred to as anal cancer, has increased steadily over the past 4 decades in the Western world [1, 2]. The standard-of-care treatment for non-metastatic SCCA is definitive chemoradiation (CRT) [3]: its aim is to eradicate the tumour while preserving anal sphincter function.

Magnetic resonance imaging (MRI) is recommended in Europe as the imaging modality of choice for loco-regional staging of SCCA [3] and has a growing role in radiation therapy planning [4]. High-resolution T_2_-weighted (T_2_-w) sequences, obtained in the appropriate planes, provide detailed anatomical depiction of the anorectal region thanks to optimal soft-tissue contrast [5–8] and are in principle best suited for accurate target volume delineation.

Diffusion-weighted imaging (DWI) is now routinely included in body MRI protocols in most European oncological imaging centres: it has been shown to aid the diagnosis and response assessment of a variety of malignancies [9–13] and to allow the detection of small tumours in the pelvis [14]. Hypercellular tumours restrict water diffusion in the extracellular-extravascular space and typically stand out as bright lesions on a ‘dark’ background of suppressed signal on high *b*-value sequences, facilitating detection and delineation. Anal cancers typically appear restricted on DWI [15].

Maximum tumour diameter (MTD) is an important measurement in anal cancer, as it determines the T stage according to current TNM (7^th^ ed.) criteria [16] (Table 1). Gross tumour volume (GTV), defined as the gross primary anal tumour volume, forms the basis to calculate clinical and planning target volumes, which in turn determine radiotherapy dose distribution. Accurate GTV delineation is critical to the delivery of intensity-modulated radiotherapy (IMRT), which produces steep dose gradients and allows dose escalation to smaller high-risk target volumes (simultaneous integrated boost radiotherapy, SIBR) [17].

**Table 1:** SCCA primary tumour (T) staging criteria according to the AJCC Cancer Staging Manual, 7th edition [16]

TX	Primary tumour cannot be assessed
T0	No evidence of primary tumour
Tis	Carcinoma in situ
T1	Tumour ≤2 cm in greatest dimension
T2	Tumour >2 cm and ≤5 cm in greatest dimension
T3	Tumour >5 cm in greatest dimension
T4	Tumour of any size invading adjacent organs

This study aimed to investigate the extent to which MRI measurements, specifically MTD and GTV, differ between anatomical T_2_-w and functional DWI sequences, as the implications for staging and treatment planning are clearly relevant to clinical practice. Second, it aimed to measure interobserver agreement for MTD and GTV as well as compare tumour detection confidence between observers with differing levels of interpretation experience.

## Materials and methods

A review board waiver was granted for this retrospective analysis of anonymised imaging data acquired as part of normal clinical care. Fifty patients with biopsy-proven SCCA undergoing pelvic MRI for locoregional staging prior to definitive chemoradiation were identified from the picture archiving and communication system (PACS) of two tertiary-referral cancer centres, between July 2007 and June 2015. Cases were excluded if the tumour was incompletely imaged on either T_2_-w sequences or DWI (*n* = 3); the primary tumour was deemed undetectable on either sequence by secondary consensus reading (*n* = 2); the presence of MRI image artefact precluded accurate tumour measurements (*n* = 0).

### Imaging protocol

Patients were scanned supine on one of three 1.5-T MRI scanners (Magnetom Avanto or Aera, Siemens Healthineers, Erlangen, Germany) using a pelvic phased array coil. The examination protocol included a T_2_-w sagittal turbo spin echo (TSE) sequence covering the pelvis (typical acquisition parameters: TR/TE = 4430/100 ms, NEX = 2, ST = 3 mm, gap 0.3 mm, FOV 250 × 250 mm, matrix = 307 × 384), a T_1_-w axial TSE sequence for pelvic nodal detection (TR/TE = 552/11 ms, NEX = 1, ST = 5 mm, gap = 1.5 mm, FOV = 300 × 300 mm, matrix = 240 × 320), a T_2_-w axial TSE sequence of the pelvis (TR/TE = 4590/101 ms, NEX = 1, ST = 5 mm, gap = 1.5 mm, FOV = 300 × 300, matrix = 307 × 384) and high-resolution small-field-of-view T_2_-w TSE sequences perpendicular and parallel to the anal canal (TR/TE = 6530/104 ms, NEX = 2, ST = 3 mm, gap = 0.3 mm, FOV = 200 × 200, matrix = 512 × 512). DWI consisted of a single shot spin echo-echo planar imaging (SE-EPI) axial diffusion-weighted sequence (TR/TE = 5900/68 ms, NEX = 4, ST = 5 mm, gap = 1.5 mm, FOV = 300 × 300, matrix = 116 × 154) encompassing the pelvis with three *b*-values in all cases (0, 100, 800 s/mm^2^). Vendor-generated apparent diffusion coefficient (ADC) maps were automatically created at the time of acquisition. Patients did not undergo any additional preparation prior to the examination.

### MTD and GTV measurements

**Fig. 1:**
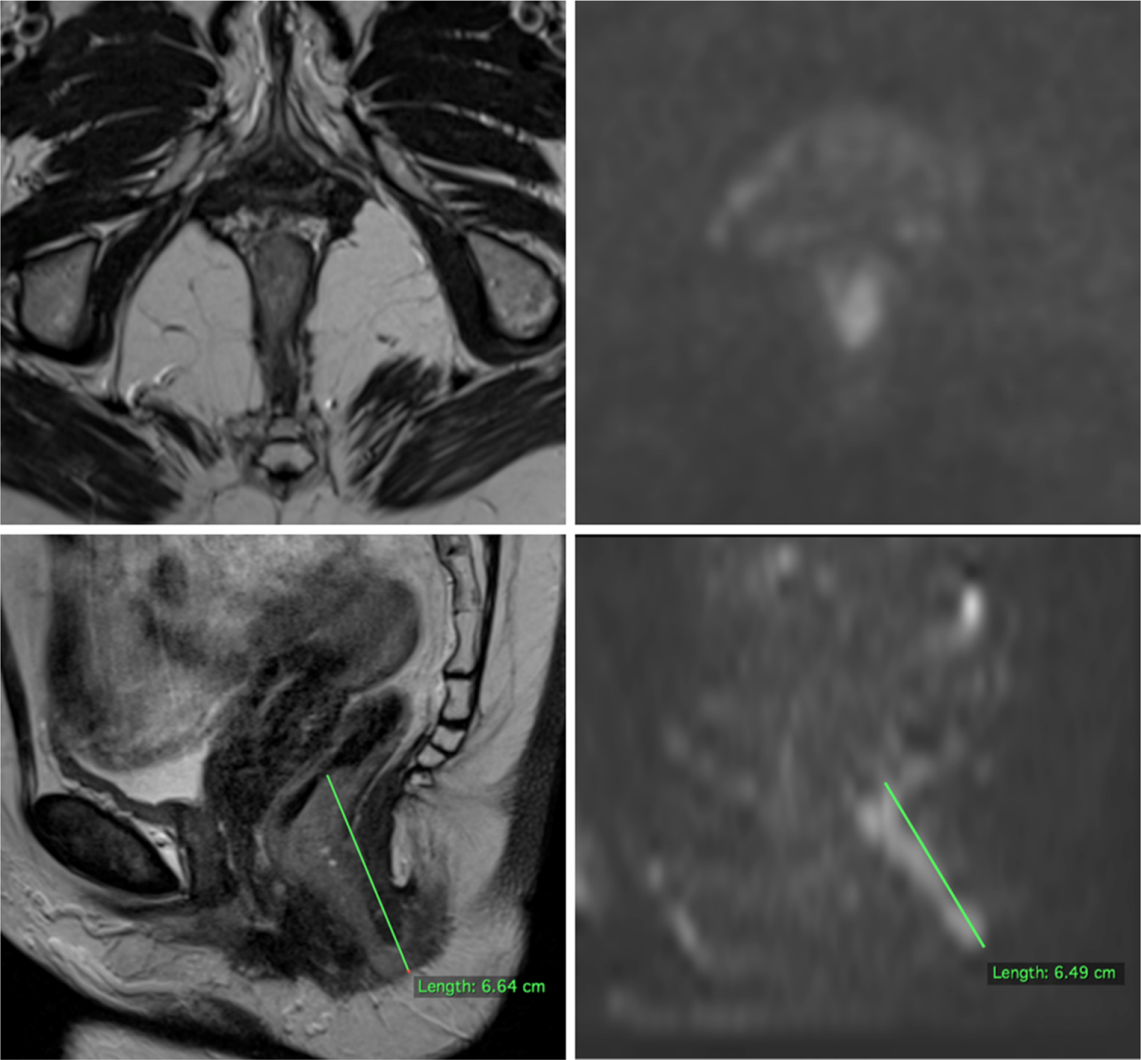
Anorectal SCCA. High-resolution T_2_-w axial-oblique image at the level of the anorectal junction (top left): lesion contouring is challenging considering the suboptimal contrast resolution between tumour and adjacent mucosa. Same-level high *b*-value axial DWI (top right): the hyperintense lesion can be clearly outlined against the suppressed signal of surrounding healthy tissue. T_2_-w sagittal image used for MTD measurement (bottom left). Corresponding sagittal reformat of high *b*-value DWI, also used for MTD measurement (bottom right)

A third-year radiology resident (RM) with 1 year prior MRI experience but no previous experience in staging SCCA and a subspecialty gastrointestinal radiology fellow (DP) with 5 years’ experience of staging gastrointestinal cancers evaluated the scans independently using all available sequences. Anonymised scans were downloaded from the local PACS onto a standalone workstation (iMac®, Apple Inc., CA, USA) and presented in randomised order in OsiriX v.7.5.1 (OsiriX Foundation, Geneva, Switzerland); readers were blinded to all clinical information. GTV delineation was performed separately on high-resolution axial-oblique T_2_-w and axial high-b-value (b = 800) DWI sequences, with a 1-week interval between the two reading sessions; DWI was read in conjunction with apparent diffusion coefficient (ADC) maps. Free-hand perilesional regions of interest (ROIs) were drawn on each slice with visible tumour and GTVs obtained by computing the ROI volumes. MTDs were obtained from sagittal T_2_-w sequences and sagittal reformats of axial high-b-value DWI, choosing the plane yielding the longest measurement on a case-by-case basis and using straight-line measurements (Fig. 1).

### GTV confidence score

Each observer rated their confidence at contouring each tumour GTV on both T_2_-w and DWI sequences using a 5-point scale (1, no tumour boundaries identified with confidence; 2, tumour boundaries identified with confidence on a minority of images (< 25%); 3, tumour boundaries identified with confidence on approximately half of the images; 4, tumour boundaries identified confidently on most images (> 75%); 5, tumour boundaries identified confidently on all images).

### Statistical analysis

Statistical analysis was performed using IBM SPSS Statistics, version 23. Mean values between the two readers’ MTD and GTV measurements were used in T_2_-w vs. DWI comparisons; measurements were compared using the independent samples t-test and correlated using Pearson’s r. Interobserver agreement between the readers’ MTD and GTV measurements was assessed using the 95% Bland-Altman limits of agreement [18]. Intraclass correlation coefficients (two-way consistency model, absolute agreement type, average measures) were also calculated. A *P* value < 0.05 was taken to represent statistical significance for all analyses.

## Results

The final cohort consisted of 45 patients, 25 females and 20 males, with a mean age of 62 years (standard deviation, 12.5; range, 37-84 years) and corresponding to 45 MRI data sets analysed by each observer.

### MTD and GTV measurements

**Fig. 2:**
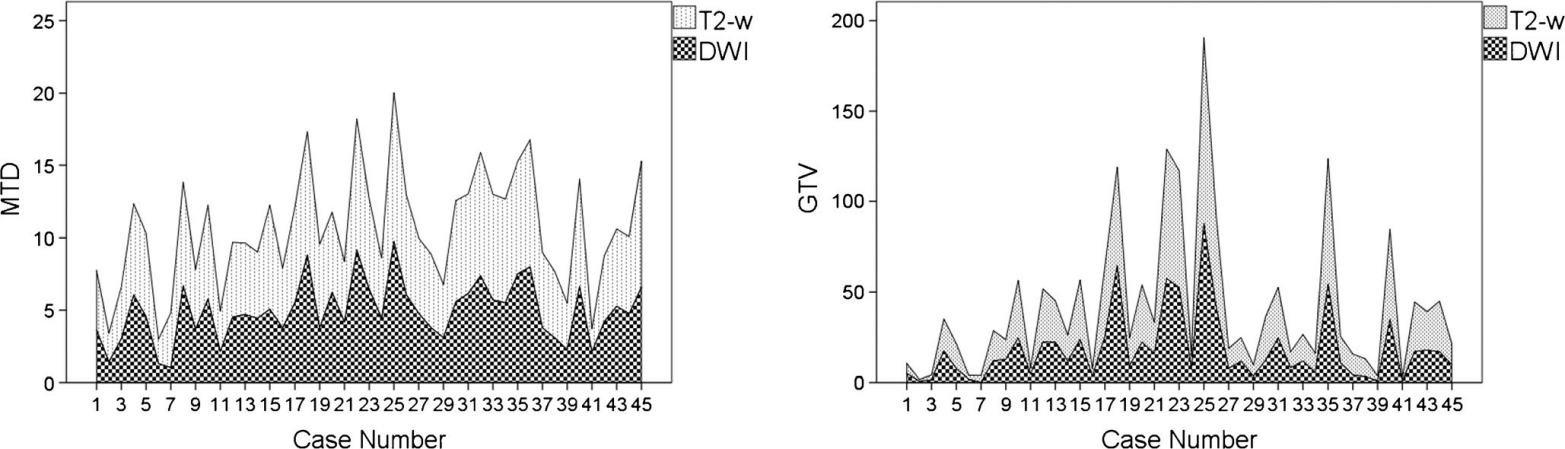
Mean MTD (cm) and GTV (cm^3^) measurements on T_2_-w versus DWI sequences, visualised case by case. Both measurements were systematically lower on DWI than on T_2_-w

**Fig. 3:**
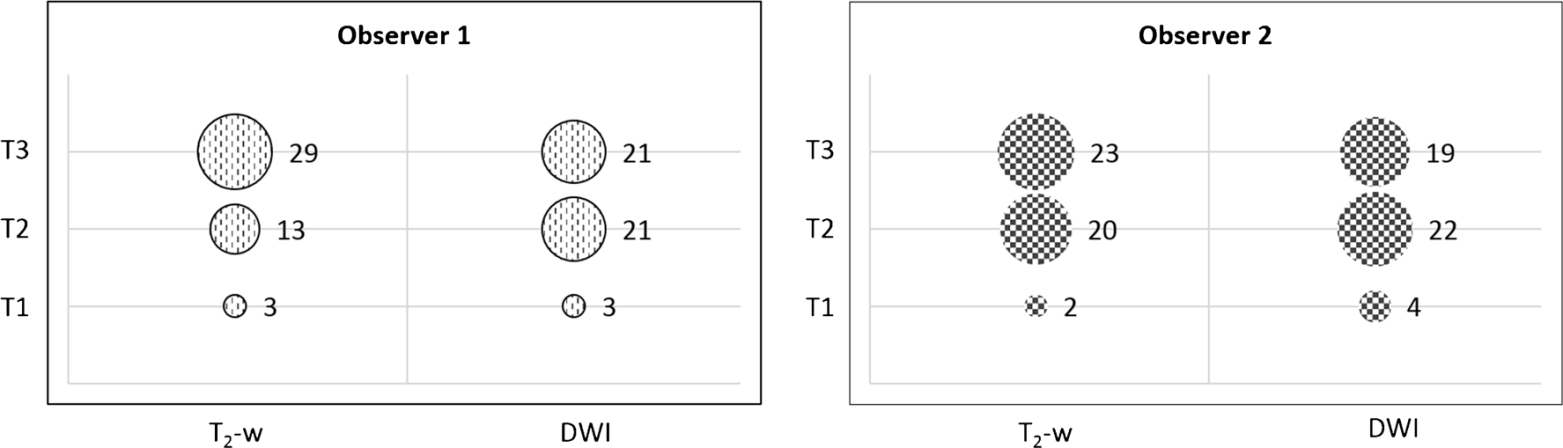
Tumour (T) staging based on MTD measurements on T2-w versus DWI. Sequence selection affects T staging, particularly when the reader is inexperienced (Observer 1)

Reader-specific tumour diameters and volumes measured on T_2_-w sequences and DWI are summarised in Table 2. GTV and MTD measurements were significantly different between T_2_-w and DWI for both observers (paired samples t-test *P* values <0.001) (Table 2) and consistently lower on DWI (Fig. 2) by percentage values ranging between 9.98% and 29.70% (Table 2). As a consequence, MTD-based tumour (T) staging was discordant in 12 cases based on inexperienced observer measurements and in 10 cases based on experienced measurements (Fig. 3). As expected, inter-sequence measurements were strongly and significantly correlated, with r values ranging between 0.875 and 0.987.

**Table 2:** Reader-specific MTD and GTV measurements (mean, standard deviation and range), paired samples t-test *P* values and Pearson correlation test r and *P* values

	Observer 1 (inexperienced)						Observer 2 (experienced)					
	MTD (cm)			GTV (cm^3^)			MTD (cm)			GTV (cm^3^)		
	Mean	SD	Range	Mean	SD	Range	Mean	SD	Range	Mean	SD	Range
T_2_-w	5.88	2.14	1.36–10.93	24.95	23.67	0.89–110.84	5.41	2.07	1.46–9.58	20.98	20.94	0.70–95.04
DWI *b*800	5.01	1.99	1.16–9.38	17.54	18.30	0.16–79.99	4.87	2.24	0.79–10.83	18.41	20.10	0.13–95.15
Relative change	−14.80%			−29.70%			−9.98%			−12.25%		
t-test *P*	<0.001			<0.001			<0.001			<0.001		
Correlation r	0.875			0.949			0.906			0.987		
Correlation *P*	<0.001			<0.001			<0.001			<0.001

### Interobserver agreement

**Fig. 4:**
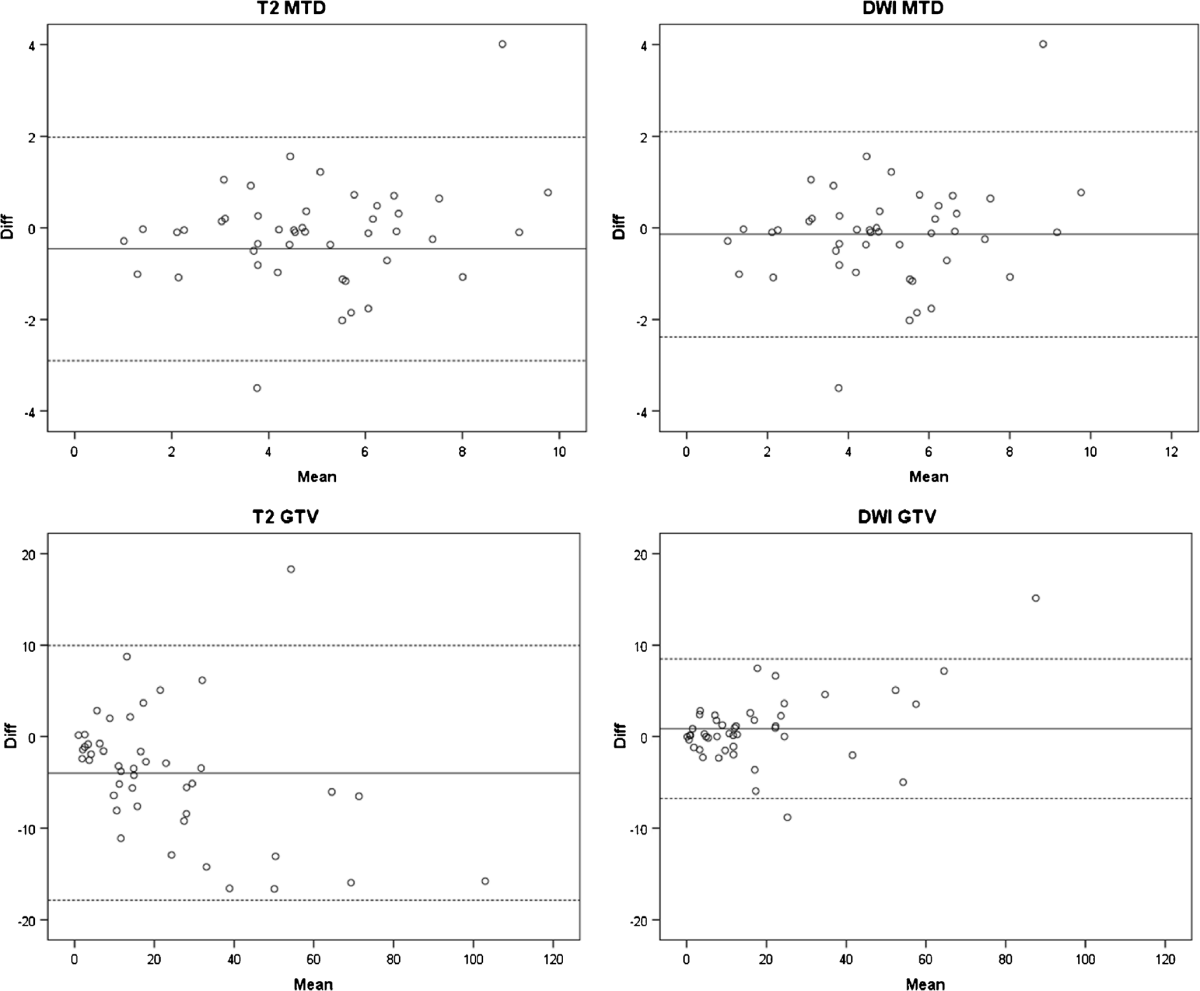
Interobserver agreement. Bland-Altman plots for MTD and GTV on T_2_-w versus DWI sequences: relative interobserver differences (mean difference and 95% limits of agreement) are plotted against the mean value

Agreement was marginally superior on DWI for MTD. Mean MTD difference (95% limits of agreement) between the two readers was -0.46 (-2.89 to +1.97) cm on T_2_-w and -0.14 (-2.38 to +2.10) cm on DWI. Agreement was considerably superior on DWI for GTV. Mean GTV difference was -3.96 (-17.91 to +9.97) cm^3^ on T_2_-w and 0.87 (-6.75 to +8.50) cm^3^ on DWI (Fig. 4). Intraclass correlation coefficients, reported in Table 3, were well above 0.8 (indicating excellent agreement) but higher for DWI.

**Table 3:** Intraclass correlation coefficients (95% confidence intervals)

	MTD	GTV
T_2_-w	0.899 (0.803–0.946)	0.968 (0.915–0.985)
DWI *b*800	0.925 (0.863–0.959)	0.990 (0.918–0.994)

### GTV confidence scoring

**Fig. 5:**
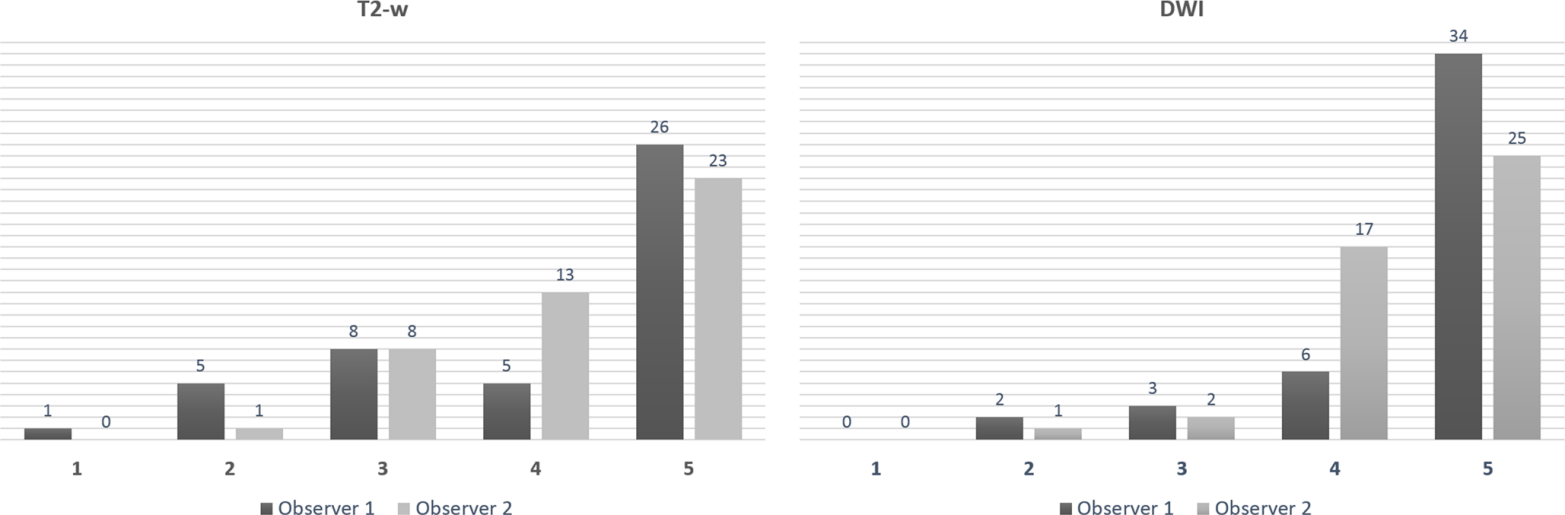
Confidence scores. Both the inexperienced (Observer 1) and the experienced reader (Observer 2) outlined tumours confidently (scores of 4 to 5) more frequently on DWI than on T_2_-w. The confidence gain with DWI is greater for the inexperienced observer

Tumours were outlined with greater confidence on DWI than on T_2_-w sequences by both readers. This gap in confidence was more substantial for the inexperienced reader: they assigned a low confidence score (1 to 3) to 14 cases on T_2_-w versus 5 cases for the experienced reader on DWI and a high confidence score to 31 cases on T_2_-w versus 40 cases on DWI. Full confidence score results are reported in Figs. 4 and 5.

## Discussion

We found that tumour volumes and maximum diameters measured on functional DWI were significantly lower than those measured on anatomical T_2_-w sequences.

While no previous paper has investigated anal cancer, similar differences have been found in primary rectal cancer [19–21]: Curvo-Semedo et al. reported median pre-treatment tumour volumes of 18.0 cm^3^ on DWI versus 21.8 cm^3^ on T_2_-w, a relative difference of 17.43%; similarly, Regini et al. measured smaller GTVs on DWI, with relative differences of 3.04% -7.62% not reaching statistical significance. Squamous cell carcinomas are typically associated with markedly impeded diffusion and display high signal intensities on DWI; adenocarcinomas, conversely, only appear moderately restricted because of their glandular structure and presence of mucin [22]. This pathological difference is likely to contribute to smaller DWI measurements in anal cancer compared to T_2_-w sequences.

Tumour greatest dimension is the only measurement determining T stage in SCCA according to AJCC TNM criteria [16]: based on our results, tumours bordering 2 cm and 5 cm in MTD (corresponding to T1/T2 and T2/T3 thresholds, respectively) are prone to categorisation variability, depending on both the reader and the sequence chosen for measurement: approximately a fourth of cases in our series were assigned a discordant T stage between T_2_-w and DWI sequences by both the inexperienced and experienced observer. With the wider implementation of personalised radiotherapy protocols, MTD and, consequently, T stage may also affect the GTV to clinical target volume (CTV) margin, the dose to the primary tumour and the use of simultaneous boost; the PLATO (Personalising Anal Cancer Radiotherapy Dose) protocol, for example, mandates an isocentric GTV-CTV margin of 10 mm for tumours up to 4 cm in MTD versus 15 mm for larger tumours [23]. To our knowledge, to date no other study has described the scale of this potential modality-, sequence- and observer-dependent variability and specific guidelines are still lacking on the matter.

Accurate tumour delineation is critical to radiotherapy planning. With the implementation of intensity modulated radiotherapy (IMRT) in the treatment of SCCA, it has become possible to escalate the dose to the target volume whilst maintaining the same or reducing dose to the surrounding normal tissues, resulting in steep dose gradients. To ensure appropriate dose delivery, both tumour and normal tissues must be delineated in 3D with high precision in reference to advanced diagnostic imaging techniques, including functional imaging [24]. The importance of access to high-quality diagnostic imaging has been illustrated by the US-based RTOG 0529 phase II trial evaluating dose-painting IMRT in SCCA, in which the gross tumour was inaccurately delineated in 21% of cases [25].

MRI is recognised in Europe as the modality of choice for locoregional staging of SCCA because of its high soft tissue contrast and its ability to depict local tumour infiltration; most clinical oncologists will refer to diagnostic MRI images at the time of planning: these can be co-registered with planning CT images used for dose calculation. The limiting factor in this setting may be the lack of experience in MRI interpretation; T_2_-w sequences represent the bedrock of pelvic MRI for detailed anatomical interpretation but require an advanced level of knowledge of the relevant cross-sectional anatomy. Signal intensities of tumour, muscle, fat and bowel contents are often very similar and can be challenging to tell apart with confidence. We believe our results partly reflect the challenges of distinguishing tumour from normal tissue in the anorectum on anatomical T2-w sequences alone. Fourteen and nine cases were assigned a low confidence score (1 to 3) by the inexperienced and experienced observer, respectively; these corresponded to either small (T1/T2) tumours with irregular margins and an infiltrative behaviour through the anal sphincter complex or anorectal junctional tumours surrounded by mucosal oedema and/or luminal fluid (Fig. 1).

In this context, the typically bright appearance of SCCA against a dark background on high b-value DWI facilitates tumour delineation based on our study results. DWI certainly improved the confidence of both the inexperienced and experienced observer in outlining tumours in this study.

A drawback of the most commonly used single-shot echo-planar-imaging (EPI)-based DWI sequence is that it is prone to artefacts and susceptibility-related geometrical distortions, potentially detrimental in the setting of radiotherapy planning. These issues are being addressed through the development of distortion-correction strategies [26] and the optimisation of turbo spin echo (TSE)-based sequences [27]. In our high-*b*-value DWI series, the most common cause for measurement discrepancies between observers was the inclusion by the inexperienced observer of susceptibility artefacts at the anal verge (tissue-air interface), emphasising the importance of taking the learning curve into account when approaching DWI.

Regarding the potential implications of underestimating vs. overestimating tumour length/volume, it is worth stressing that the current research focus in patients with early disease is radiotherapy dose de-escalation, given the low rates of locoregional failure and significant toxicity at current dose regimens [3, 23]. Conversely, patients with locally advanced disease, 30% of whom experience locoregional failure, may benefit from higher radiotherapy doses or sequential boosts by means of IMRT [28, 29]. Applying these considerations to our study series and assuming experienced measurements as ‘accurate’, six cases would have been overstaged as T3 (advanced) disease by the inexperienced observer based on T2 sequences alone; none understaged; only 2 based on DWI (Fig. 3). Complementing T2 sequences with DWI, therefore, would seem more likely to save patients from radiotherapy toxicity than compromise their outcome by size underestimation.

This study has a number of limitations: its retrospective nature meant that minor variations in the imaging acquisition across different 1.5-T scanners could not be avoided; the sequences used for measurements and DWI *b*-values were nevertheless consistent. We did not evaluate spatial concordance and volume overlap between T_2_-w and DWI, as performed by Burbach et al. for rectal cancer [30], though it would be interesting to assess the entity of geometrical distortions in anal cancer using conventional EPI-based DWI sequences. As DWI was acquired as a 2D axial sequence with a 1.5-mm slice gap, sagittal reformats yielded slightly blurred images with a potential impact on MTD measurements: it is reassuring nevertheless that the trend for smaller measurements on DWI was maintained.

In summary, this study has shown that anal cancer MTD and GTV measurements are consistently and significantly lower on DWI than on T2-w sequences, with consequent intersequence T staging discordances and potential implications for radiotherapy target volume delineation. This highlights the need for more specific guidelines on the subjects. Based on these findings and our clinical experience we would recommend the inclusion of DWI in anal cancer staging/radiotherapy planning MRI protocols and its use alongside anatomical sequences. DWI measurements resulted in higher agreement between observers with differing levels of experience. DWI offered greater tumour delineation confidence over T_2_-w sequences to the inexperienced observer and even to the experienced in the case of small tumours infiltrating the anal sphincter complex or at the anorectal junction.

## Abbreviations

DWI: Diffusion-weighted imaging
GTV: Gross tumour volume
IMRT: Intensity-modulated radiotherapy
MTD: Maximum tumour diameter
SCCA: Squamous cell carcinoma of the anus
SIBR: Simultaneous integrated boost radiotherapy
T_2_-w: T_2_ weighted
